# Kinetics in HBsAg after Stopping Entecavir or Tenofovir in Patients with Virological Relapse but Not Clinical Relapse

**DOI:** 10.3390/v14061189

**Published:** 2022-05-30

**Authors:** Tzu-Ning Tseng, Yuan-Hung Kuo, Tsung-Hui Hu, Chao-Hung Hung, Jing-Houng Wang, Sheng-Nan Lu, Chien-Hung Chen

**Affiliations:** Division of Hepatogastroenterology, Department of Internal Medicine, Kaohsiung Chang Gung Memorial Hospital, Chang Gung University College of Medicine, Kaohsiung 833, Taiwan; mice0930@cgmh.org.tw (T.-N.T.); 0104kuo@gmail.com (Y.-H.K.); dr.hu@msa.hinet.net (T.-H.H.); chh4366@yahoo.com.tw (C.-H.H.); jinghoung2001@yahoo.com.tw (J.-H.W.); juten@ms17.hinet.net (S.-N.L.)

**Keywords:** HBsAg, generalized estimating equations, entecavir, tenofovir

## Abstract

This study investigated the kinetics in HBsAg and the HBsAg loss rate after entecavir or tenofovir disoproxil fumarate (TDF) cessation in patients with chronic hepatitis B (CHB) who achieved virological suppression after virological relapse without clinical relapse. A total 504 HBeAg-negative, non-cirrhotic patients who previously received entecavir or TDF with post-treatment and who were followed up for at least 30 months were included. Of the 504 patients, 128 achieved sustained virological suppression (Group I), and 81 experienced virological relapse without clinical relapse. Of the 81 patients, 52 had intermittent or persistent HBV DNA > 2000 IU/mL (Group II), and 29 achieved persistent virological suppression (HBV DNA < 2000 IU/mL) for at least 1.5 years (Group III) after virological relapse. A generalized estimating equations analysis showed that Groups I and III experienced larger off-treatment HBsAg declines than Group II (both, *p* < 0.001). The post-treatment HBsAg declines of Group I and Group III were similar (*p* = 0.414). A multivariate analysis showed that there were no differences in the HBsAg change and HBsAg decline (*p* = 0.920 and 0.886, respectively) or HBsAg loss rate (*p* = 0.192) between Group I and Group III. The patients who achieved persistent viral suppression after HBV relapse without clinical relapse have a similar decline in HBsAg and the HBsAg loss rate as the sustained responders.

## 1. Introduction

Nucleos(t)ide analogues (NAs) have been used for patients with chronic hepatitis B (CHB). NAs can suppress hepatitis B virus (HBV) replication and reduce hepatic inflammation. However, hepatitis B surface antigen (HBsAg) loss rarely occurs during long-term NA treatment [1,2], and the frequency of HBV relapse is high after the discontinuation of NA therapy [3,4,5].

Despite HBV relapse being a frequent occurrence after NA therapy withdrawal, previous researchers have demonstrated that the rate of HBsAg loss significantly increases after the discontinuation of NA therapy [6,7,8,9]. Recent studies have demonstrated that the rate of HBsAg seroclearance was the highest in sustained responders after NA therapy cessation [6,10]. Moreover, patients who did not require retreatment after clinical relapse had a higher rate of HBsAg loss than those who required retreatment [6,10]. Our previous study demonstrated that, at 5 years after NA cessation, the cumulative probability of HBsAg seroclearance was 12.8% among 37 patients who experienced virological relapse, but not clinical relapse, following the withdrawal of entecavir treatment [10]. Therefore, some patients in this group could achieve persistent virological suppression, a subsequent decline in HBsAg levels, or even HBsAg loss after virological relapse, although ALT flares did not occur in these patients. Thus, the investigation of the incidence and predictors of persistent virological suppression in patients with virological relapse but not clinical relapse is merited. Furthermore, it remains unclear whether the decline in the HBsAg levels and HBsAg loss rate for patients with virological relapse but not clinical relapse is similar to that of sustained responders. The aims were to study the incidence and predictors of persistent virological suppression after virological relapse without clinical relapse and compare the change in HBsAg and the HBsAg loss rate between HBeAg-negative patients with persistent virological relapse after virological relapse and patients with a sustained response after the withdrawal of entecavir or tenofovir disoproxil fumarate (TDF) therapy.

## 2. Materials and Methods

### 2.1. Patients

A total of 330 CHB patients who received entecavir treatment from 2008 to 2017 and 174 CHB patients who received TDF treatment from 2011 to 2017 were enrolled. The followed-up duration off-therapy was at least 30 months in all patients. All patients were positive for HBsAg for at least 6 months before treatment and were HBeAg-negative and non-cirrhotic before the NA treatment. All patients fulfilled the practice stopping guideline of the Asian Pacific Association for the Study of the Liver 2012 [11]. The National Health Plan of Taiwan reimbursed the cost of NA therapy for 3 years for the HBeAg-negative non-cirrhotic patients during the study period. The individual patients and physicians determined whether NA therapy should be prolonged according to the patient’s willingness.

The absence of cirrhosis was determined by either a biopsy (*n* = 26) or a repeated ultrasound showing the absence of cirrhosis [12]. The definition of virological relapse was HBV DNA levels > 2000 IU/mL Clinical relapse was a combination of an alanine aminotransferase (ALT) > 2, the upper limit of normal, and an HBV DNA level > 2000 IU/mL after NA withdrawal [11]. Of the 504 patients, 128 achieved a sustained virological suppression (HBV DNA < 2000 IU/mL) during post-treatment follow-up (median: 241 weeks (range: 135–516)) until the last visit (Group I), and 81 experienced virological relapse without clinical relapse during the post-treatment follow-up until the last visit (median: 225 weeks (range: 132–588) after the cessation of entecavir or TDF therapy). All of these 81 patients had a follow-up for at least 1.5 years after the first virological relapse. We divided the 81 patients who experienced virological relapse but not clinical relapse into two subgroups based on their relapse patterns: Group II, 52 patients who had persistent or intermittent HBV DNA > 2000 IU/mL for at least 1.5 years after virological relapse until the last visit; and Group III, 29 patients with persistent virological suppression (HBV DNA < 2000 IU/mL for at least 1.5 years until the last visit) after virological relapse. These 209 patients did not receive retreatment and were enrolled in this study for analysis. Of the 295 patients who did not belong to Groups I, II and III, 60 experienced clinical relapse without retreatment, and 235 received retreatment of NA during the off-NA follow-up.

The exclusion criteria were (1) hepatitis C virus, hepatitis D virus or human immunodeficiency virus co-infection; (2) cirrhosis or hepatocellular carcinoma before or during treatment; (3) alcoholic liver disease or autoimmune hepatitis. (4) HBsAg loss during treatment; (5) patients who received immunosuppressive therapy.

### 2.2. Methods

After entecavir or TDF cessation, the liver function test and HBV seromarkers were monitored every 1–3 months within the first 12 months and then every 3–6 months. During the increase of alanine aminotransferase (ALT) levels > 1× ULN, additional monitoring was performed for close monitoring of the early signs of hepatic decompensation. Serum HBV DNA was tested every 1–3 months within the first 6 months and every 3–6 months thereafter. Additional HBV DNA monitoring was performed if the patients experienced virological relapse. HBsAg was quantified retrospectively at the time of the initial treatment and at the end of treatment (EOT). Serum HBcrAg was determined at the time of the initial treatment and the EOT of entecavir or TDF therapy.

### 2.3. Serological and Virological Testing

Serum HBsAg titers were determined using the Roche Elecsys HBsAg II Quant reagent kit (Roche Diagnostics, Indianapolis, IN, USA), with a detection limit of 0.05 IU/mL. Serum HBV DNA was assayed using the COBAS TaqMan HBV monitoring test (Roche Diagnostics, Branchburg, NJ, USA), with a detection limit of 20 IU/mL.

Serum HBcrAg was quantified using the Lumipulse G HBcrAg assay on a LUMIPULSE G1200 Analyzer (Fujirebio Inc., Tokyo, Japan) [13]. The detection linear range of this array was 3 to 7 log U/mL. HBcrAg levels below 3 log U/mL were considered negative. Thus, an HBcrAg level blow 3 log U/mL was classified as 3 log U/mL for the statistical analysis. Serum HBcrAg levels above 7 log U/mL were diluted with a dilution reagent in order to determine the final titer.

### 2.4. HBV Genotyping

The genotypes of HBV were performed by restriction fragment length polymorphism (RFLP) [14]. The direct sequencing was used if the RFLP method could not determine the kind of genotype.

### 2.5. Statistical Analysis

A comparison between the different groups was performed using the Chi-square or Student’s *t* test. The cumulative incidence of HBsAg loss was calculated with the Kaplan–Meier method and compared using the log-rank test. The risks of HBsAg loss were evaluated between Groups I and III using the Cox proportional hazard regression model. The significant factors in the univariate analysis were included in the multivariate analysis. *p* values < 0.05 were considered to be significant.

The generalized estimating equations (GEEs) were analyzed for changes or declines in the HBsAg levels during or after NA therapy between Groups I, II and III. A multivariate analysis of the GEE model was used through an exchangeable working correlation.

## 3. Results

### 3.1. Clinical Features of Patients with Sustained Virological Suppression and Patients with Virological Relapse but No Clinical Relapse

The clinical characteristics of the patients who achieved a sustained virological suppression and the patients who experienced virological relapse but not clinical relapse are compared in Table 1. The patients with a sustained virological suppression had a higher rate of TDF treatment and had lower levels of HBsAg and HBV DNA at baseline and of HBsAg at EOT than the patients with virological relapse without clinical relapse.

### 3.2. Clinical Features of Patients with and without Persistent Virological Suppression after Virological Relapse

The 81 patients who developed virological relapse but not clinical relapse were stratified into two groups: Group II, persistent or intermittent virological relapse after virological relapse; and Group III, persistent virological suppression after virological relapse. The clinical features of the two groups are presented in Table 2. No significant difference was noted in any parameter at baseline or at EOT between Group II and Group III. However, Group III had lower HBsAg levels at the time of the first virological relapse than Group II. There was no significant difference in the FIB-4 index between Group I and Group III (mean: 3.54 ± 3.57 vs. 2.61 ± 2.07, *p* = 0.271).

### 3.3. Comparison of HBsAg Changes between Groups I, II and III

At the initial therapy, Group I had lower HBsAg levels than Group II (2.54 ± 1.03 versus 2.95 ± 0.64 log IU/mL, *p* = 0.009). At the initial treatment, no significant differences were presented in the HBsAg levels between Groups I and III (*p* = 0.097) or between Groups II and III (*p* = 0.695). At the end of therapy, Group I had lower HBsAg levels than Group II (1.68 ± 1.05 versus 2.61 ± 0.64 log IU/mL, *p* < 0.001) and Group III (1.68 ± 1.05 versus 2.34 ± 0.80 log IU/mL, *p* = 0.002). The HBsAg levels in Group II were similar to those in Group III (*p* = 0.091).

**Figure 1:**
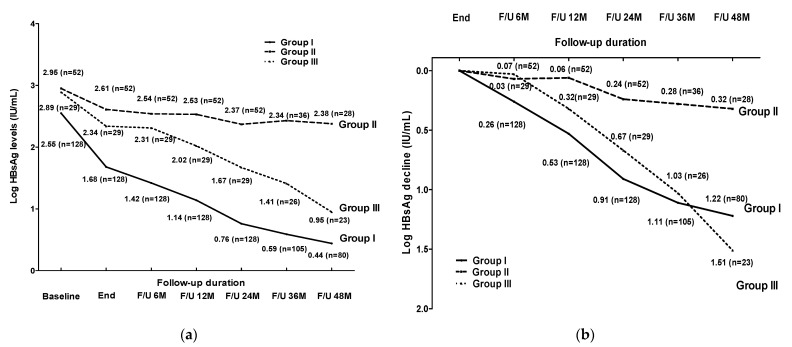
HBsAg kinetics (**a**) and HBsAg decline (**b**) after entecavir or TDF cessation, stratified by the different relapse pattern. Group I: 128 patients with a sustained virological suppression; Group II: 52 patients with persistent or intermittent virological relapse; Group III: 29 patients with a persistent virological suppression after virological relapse.

GEE analysis was employed to estimate the correlations between the HBsAg kinetics of the different groups. Groups I and III had larger HBsAg changes than Group II (*p* < 0.001 and *p* = 0.003, respectively). Group I had larger HBsAg changes than Group III (*p* = 0.001; Figure 1a).

We also used GEE analysis to evaluate the correlations between the post-treatment HBsAg declines at EOT across the groups. Group I and Group III exhibited larger HBsAg declines than Group II (both *p* < 0.001). The post-treatment HBsAg declines of Group I and Group III were similar (*p* = 0.414; Figure 1b). There were no significant differences in the post-treatment HBsAg changes and declines between Groups I and III (*p* = 0.92 and 0.886, respectively) after adjusting for various clinical features and HBcrAg and HBsAg levels (Table 3 and Table 4).

We compared the HBsAg kinetics and declines between the entecavir and TDF groups. For all 209 patients, there was no significant difference in HBsAg decline during treatment (from initial treatment to end of treatment) between the entecavir and TDF groups (0.64 ± 0.84 versus 0.79 ± 0.88 log IU/mL, *p* = 0.223). Furthermore, no differences were noted in the HBsAg changes and declines between the entecavir and TDF groups for all patients (*p* = 0.124 and *p* = 0.318, respectively) in Group I (*p* = 0.279 and *p* = 0.405, respectively) and in Groups II and III (*p* = 0.394 and *p* = 0.270, respectively), according to the GEE analysis. In the patients in Groups II and III, the time of the virological relapse was no different between the entecavir and TDF groups (63.4 ± 73.7 versus 40.7 ± 31.6 weeks, *p* = 0.188).

### 3.4. HBsAg Seroclearance in Groups I and III

**Figure 2:**
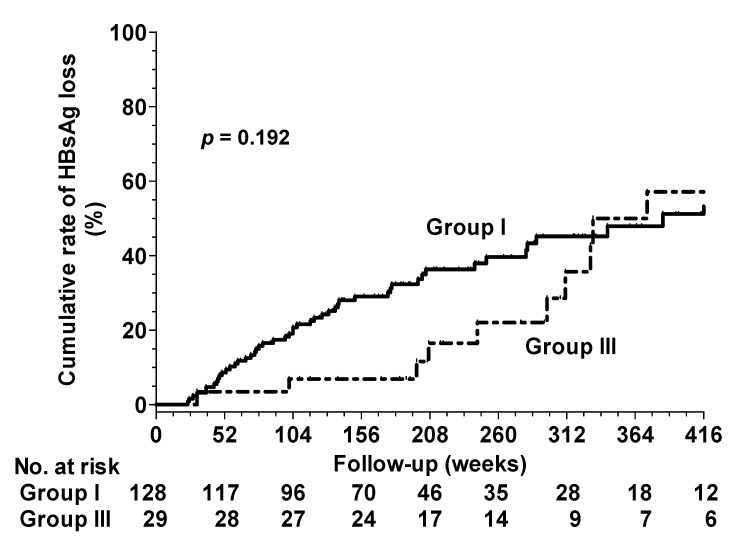
HBsAg loss rates between Group I and Group III.

Among the 128 patients in Group I and the 29 patients in Group III, 52 and 11 developed HBsAg loss, respectively. The time to the HBsAg loss was longer in Group III than that in Group I (272.8 ± 135.2 versus 197.7 ± 123.6 weeks, *p* = 0.004). However, the cumulative incidences of HBsAg loss at 8 years in Groups I and III were 51.2% and 57.1%, respectively. The rate of HBsAg loss in Group I was similar to that of Group III (*p* = 0.192; Figure 2). The multivariate analysis demonstrated that the lower HBsAg levels at EOT and the longer duration of the treatment were independent factors associated with HBsAg loss (Table 5). No patient in Group II exhibited post-treatment HBsAg loss.

### 3.5. Clinical Features, HBsAg Decline and HBsAg Loss in Patients with ALT Flare without Retreatment

**Figure 3:**
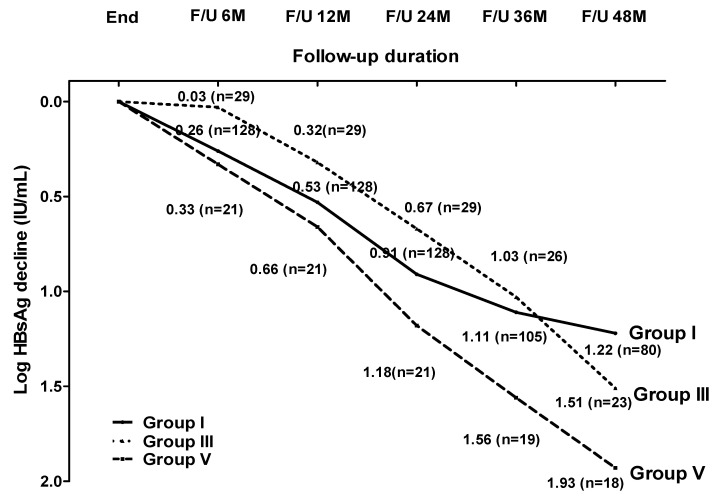
HBsAg decline stratified by the different relapse pattern. Group I: 128 patients with a sustained virological suppression; Group III: 29 patients with persistent virological suppression after virological relapse; Group V: 21 patients with persistent virological suppression after clinical relapse.

The clinical features of the patients who achieved a sustained virological suppression and the patients who experienced clinical relapse without retreatment are compared in Table 6. The patients with a sustained virological suppression had a higher rate of HBV genotype C and had lower baseline HBV DNA levels and EOT HBsAg levels than the patients with clinical relapse without retreatment. Of the 60 patients with clinical relapse without retreatment, 39 experienced persistent or intermittent virological relapse after clinical relapse (Group IV), and 21 achieved persistent virological suppression after clinical relapse (Group V). The GEE analysis showed that the HBsAg decline in Groups I and V (*p* = 0.141) and that in Groups III and V (*p* = 0.078) were similar (Figure 3). The cumulative rates of HBsAg loss at 8 years in Group V were 62.7%, There was no difference in terms of the HBsAg loss rates between Groups I and V (*p* = 0.768) and between Groups III and V (*p* = 0.478).

## 4. Discussion

In this study, 128 (25.4%) of the 504 patients achieved persistent virological suppression and 81 (16.1%) experienced virological relapse but not clinical relapse. Our previous study demonstrated that, out of 250 patients who discontinued entecavir, 71 (28.4%) had persistent virological suppression and 35 (15%) developed virological relapse but did not develop clinical relapse or require retreatment [7]. Another study of 691 patients who discontinued NA therapy showed that 144 (20.8%) did not have virological relapse and 128 (18.5%) had virological relapse but not clinical relapse [6]. The relapse rates of the two groups in this study are consistent with those of previous studies [6,7]. However, the rate of persistent virological suppression after virological relapse in the patients with virological relapse but not clinical relapse has rarely been reported. In this study, 29 (35.8%) of 81 patients achieved persistent virological suppression after virological relapse. These results raise two important points. Firstly, around 35% of patients could achieve persistent virological suppression after HBV flare but not ALT flare. Secondly, how can beneficial virological relapse be differentiated from a detrimental relapse?

Unfortunately, no factor at the baseline or EOT of entecavir or TDF therapy could significantly predict persistent virological suppression after virological relapse in these patients. During the off-therapy follow-up, the patients who achieved virological suppression (Group III) had lower HBsAg levels at the time of virological relapse than the patients without virological suppression (Group II). However, no optimal HBsAg level at the time of virological relapse would predict persistent virological suppression. Thus, only HBV DNA and HBsAg levels, monitored after virological relapse (without clinical relapse), could distinguish the two groups.

Previous studies showed that the rate of HBsAg seroclearance was the highest in patients with sustained virological suppression after stopping NA therapy [6,10]. Previous studies also found that patients who do not require retreatment after clinical relapse develop higher HBsAg loss rates than patients who received retreatment [6,10]. However, post-treatment HBsAg changes or declines have rarely been reported in patients who achieve viral suppression after virological relapse without clinical relapse. In this study, patients with persistent viral suppression after virological relapse had similar HBsAg changes and declines compared to those with sustained virological suppression after stopping NA therapy. One previous study demonstrated that the HBV-specific T-cell response in the host may be a recovery after the long-term suppression of HBV by NA therapy [15]. However, the efficient immune response must be triggered by the re-exposure to the antigens of HBV [16]. Virological relapse occurs frequently after stopping NA therapy and may induce an immune response against HBV. Thus, patients with an “effective immune response” still exhibit a subsequent decrease in HBsAg levels after an HBV flare without an ALT flare [17,18]. HBV clearance by the immune system required cytotoxic CD4+ and CD8+ T lymphocytes to kill infected cells by the virus. Cytotoxic T cells could inhibit HBV by killing infected cells but could also suppress the virus by secreting cytokines, such as IFN-γ and TNF-α, that inhibit HBV replication via a non-cytolytic mechanism [19,20,21,22]. In this study, among the patients who developed persistent virological suppression after virological relapse without clinical relapse, HBV may be suppressed through noncytolytic immune mechanisms. On the contrary, the HBsAg levels at EOT in Group I were lower than those in Group III. The reduction in the HBsAg levels potentially restored anti-HBV immune responses. Previous studies showed that increased frequencies of functional HBV-specific CD8+ T cells at EOT correlate with sustained viral control off-treatment, with the absence of HBV reactivation [23,24]. This means that the host immunity had been modulated during the long period of NA treatment and become more effective to suppress HBV replication in Group I [18,25]. This was reasonable to explain why no significant difference was presented in the post-treatment HBsAg changes and declines between Groups I, III and V. The patients who had viremia and ALT levels < 2× ULN represent a “grey zone” for antiviral treatment. We suggest that, instead of immediate retreatment, the ALT, HBV DNA and HBsAg levels should be carefully monitored in patients who experience virological relapse but not clinical relapse after discontinuing NA therapy.

Another important issue is whether the HBsAg loss rate in patients who achieve persistent viral suppression after virological relapse is similar to that of patients who have a sustained virological suppression. Previous studies showed that the T cells are less exhausted and display a higher proliferative capacity after discontinuing NA therapy in patients that subsequently achieve HBsAg loss [24,26]. In addition, the augmentation of the natural cytotoxic responses of the NK cells was associated with HBsAg seroclearance after discontinuing NA treatment [27]. We found no significant differences in the HBsAg loss rates at 8 years between the patients in Groups I and III. The patients in Group III may exhibit a similar T cell immune response to those in Group I after triggering active HBV replication, which accelerates HBsAg decline and loss. However, the duration of HBsAg loss was longer in Group III than in Group I (mean duration: 272.8 ± 135.2 versus 197.7 ± 123.6 weeks, *p* = 0.004). This may be explained by the higher HBsAg level at EOT in Group III and by the fact that most patients experienced virological relapse within the first 4 years (28/29) after the cessation of entecavir or TDF. After triggering an effective immune response by the HBV flare, the patients in Group III achieved larger HBsAg declines after virological relapse, followed by HBsAg loss. A similar phenomenon was also observed in the patients with persistent virological suppression after clinical relapse (Group V). T-cell activation could benefit in HBsAg decline or loss. However, severe hepatitis due to HBV reactivation—and even hepatic decompensation or mortality—following NA withdrawal has been reported [8,28]. Thus, close monitoring after NA withdrawal for timely retreatment is needed to reduce the risks of hepatic decompensation and mortality induced by severe hepatitis.

Comparisons of post-treatment HBsAg kinetics and declines after entecavir or TDF withdrawal have been rarely reported. In our study, there was no significant difference in the post-treatment HBsAg change and decline between the patients who discontinued entecavir and TDF therapy among all subgroup patients.

Tanaka E et al. used HBsAg and HBcrAg levels at EOT to stratify CHB patients into three groups (low-risk, medium-risk and high-risk) in order to predict sustained virological response after the discontinuation of NA [29]. The rates of sustained virological response at 2 years were 80–90%, 50% and 10–20% in the low-, medium- and high-risk groups, respectively. Therefore, the discontinuation NA can be considered in the low-risk group, and continuous NA treatment is recommended in the high-risk group. In our study, the rates of sustained virological response at 2 years were 80%, 64.8% and 63.5% in the low-, medium- and high-risk groups, respectively, for all 209 patients (Groups I + II + III). In our study, only patients with sustained virological suppression and patients with virological relapse without clinical relapse were included. Thus, the sustained virological response rates were high in the medium- and high-risk groups. Our study found that the sustained virological response rate at 2 years was 80% in the low-risk group, which was consistent with this previous study [29]. Therefore, further studies are needed to validate the practicality of this risk stratification after NA withdrawal.

There are some limitations. Firstly, this was a retrospective and one-single-center study. Further, multi-center and prospective studies are needed to confirm our findings. Secondly, the number of patients with virological relapse with or without clinical relapse was limited. Thirdly, the population of this study only consisted of Asians, and the HBV genotypes B or C were predominant. It remains unclear whether the same HBsAg decline or HBsAg loss rate would be observed between Groups I, III and V for different ethnicities and HBV genotypes. Fourthly, the follow-up schedule of the serum HBV DNA varied from 1–3 months within the first 12 months following the discontinuation of NA therapy and every 3–6 months thereafter. The patients would have been followed for different visit times, and perhaps the appearance of the virological relapse was transient. This may have led to missed events of HBV relapse and misclassification, particularly in the group without virological relapse.

## 5. Conclusions

About 35% of patients could achieve persistent virological suppression after virological relapse without clinical relapse. The patients who achieved persistent viral suppression after virological relapse had a similar decline in HBsAg levels and HBsAg loss rates as the patients who exhibited a sustained virological suppression after entecavir or TDF withdrawal. Thus, instead of immediate retreatment, a wait-and-watch strategy was suggested to increase the decline in HBsAg levels and HBsAg loss rates in patients who achieved virological suppression after virological relapse with or without clinical relapse. Retreatment might be considered in patients who experienced intermittent or persistent high viremia for a long time after virological or clinical relapse.

## Figures and Tables

**Table 1:** Clinical features of the study population.

Variable Mean ± SD or N (%)	Patients with Sustained Virological Suppression (*n* = 128)	Patients with Virological Relapse but No Clinical Relapse (*n* = 81)	*p* Value
Age (years)	50.7 ± 10.7	51.0 ± 11.1	0.834
Sex (male vs. female)	104:24	62:19	0.412
Entecavir:TDF	76:52	61:20	0.018
ALT (U/L)	305.8 ± 422.3	257.9 ± 264.8	0.361
Total bilirubin (mg/dL)	2.45 ± 4.46	1.58 ± 1.71	0.081
NA-naive	89 (69.5%)	56 (69.1%)	0.952
HBV DNA (log IU/mL)	4.97 ± 1.80	5.73 ± 1.31	0.001
HBV genotype	-	-	0.094
B	84 (65.6%)	62 (76.5%)	-
C	44 (34.4%)	19 (23.5%)	-
FIB-4 index	3.35 ± 3.57	2.61 ± 2.22	0.098
HBsAg at baseline (log_10_ IU/mL)	2.54 ± 1.03	2.93 ± 0.70	0.004
HBsAg at EOT (log_10_ IU/mL)	1.67 ± 1.05	2.51 ± 0.71	<0.001
HBcrAg at baseline (log_10_ IU/mL)	4.57 ± 1.60	4.89 ± 1.60	0.175
HBcrAg < 3 at baseline (log_10_ IU/mL)	38 (29.6%)	16 (19.7%)	0.121
HBcrAg at EOT (log_10_ U/mL)	3.30 ± 0.59	3.32 ± 0.71	0.845
HBcrAg < 3 at EOT (log_10_ U/mL)	75 (58.6%)	45 (55.5%)	0.739
Treatment duration (weeks)	169.8 ± 52.6	167.3 ± 44.4	0.725
Consolidation duration (weeks)	141.9 ± 55.0	137.8 ± 46.0	0.581

ALT, alanineaminotransferase; EOT, end of treatment; HBV, hepatitis B virus; HBsAg, hepatitis B surface antigen; HBcrAg, hepatitis B core related antigen; NA, nucleoside analogue; TDF, tenofovir disoproxil fumarate.

**Table 2:** Clinical features of patients without (Group II) and with (Group III) persistent virological remission after virological relapse without clinical relapse.

Variable Mean ± SD or N (%)	Group II (*n* = 52)	Group III (*n* = 29)	*p* Value
Age (years)	51.6 ± 10.9	50.0 ± 11.5	0.530
Sex (male vs. female)	40:12	22:7	0.914
Entecavir:TDF	38:14	23:6	0.533
ALT (U/L)	240.0 ± 205.3	290.0 ± 349.1	0.419
Total bilirubin (mg/dL)	1.79 ± 1.99	1.21 ± 0.95	0.143
NA-naïve status	39 (75%)	17 (58.6%)	0.126
HBV DNA (log IU/mL)	5.79 ± 1.24	5.61 ± 1.43	0.538
HBV genotype	-	-	0.512
B	41 (78.8%)	21 (72.4%)	-
C	11 (21.2%)	8 (17.6%)	-
FIB-4	2.62 ± 2.32	2.61 ± 2.07	0.98
HBsAg at initial treatment (log_10_ IU/mL)	2.95 ± 0.64	2.89 ± 0.82	0.695
HBsAg at EOT (log_10_ IU/mL)	2.61 ± 0.64	2.34 ± 0.80	0.091
HBcrAg at initial treatment (log_10_ U/mL)	4.91 ± 1.52	4.83 ± 1.75	0.833
HBcrAg at EOT (log_10_ U/mL)	3.23 ± 0.46	3.50 ± 1.01	0.125
Treatment duration (weeks)	162.3 ± 23.2	176.3 ± 67.2	0.173
Consolidation duration (weeks)	129.6 ± 27.3	142.5 ± 65.8	0.061
Time to first VR from EOT (weeks)	50.2 ± 54.9	71.4 ± 82.5	0.169
HBV DNA at the first VR (log_10_ IU/mL)	4.17 ± 0.59	4.13 ± 0.51	0.761
HBsAg at the first VR (log_10_ IU/mL)	2.52 ± 0.73	2.14 ± 0.80	0.035
HBsAg decline from EOT to the first VR	0.10 ± 0.23	0.20 ± 0.29	0.096

ALT, alanine aminotransferase; EOT, end of treatment; HBV, hepatitis B virus; HBsAg, hepatitis B surface antigen; HBcrAg, hepatitis B core related antigen; NA, nucleoside analogue; VR, virological relapse; TDF, tenofovir disoproxil fumarate.

**Table 3:** Multivariable analysis of post-treatment HBsAg change by generalized estimating equations analysis.

Post-Treatment HBsAg Kinetics				
HBsAg Kinetics	Beta	Standard Error	95% CI	*p* Value
Groups				
I	Ref			
II	0.336	0.084	0.171–0.501	<0.001
III	−0.014	0.142	−0.293–0.264	0.92
Time (per month)	−0.023	0.002	−0.026–0.019	<0.001
Age (per year)	0.007	0.005	−0.002–0.016	0.132
Sex (male vs. female)	0.006	0.099	−0.188–0.2	0.954
TDF vs. entecavir	−0.031	0.097	−0.222–0.159	0.746
HBV genotype (C vs. B)	−0.146	0.101	−0.344–0.051	0.146
NA-naïve (yes vs. no)	0.189	0.098	−0.004–0.382	0.055
GPT (per U/L)	−0.00018	0.00011	−0.0004–0.00003	0.096
HBV DNA (per log IU/mL)	−0.017	0.037	−0.089–0.054	0.639
Treatment duration (per week)	−0.004	0.003	−0.009–0.002	0.233
Consolidation duration (per week)	0.001	0.003	−0.004–0.007	0.603
HBsAg at initial treatment (per log IU/mL)	−0.076	0.058	−0.189–0.037	0.189
HBcrAg at initial treatment (per log U/L)	0.089	0.032	0.027–0.151	0.005
HBsAg at EOT (per log IU/mL)	1.15	0.054	1.045–1.256	<0.001
HBcrAg at EOT (per log U/L)	0.084	0.051	−0.016–0.184	0.1

ALT, alanine aminotransferase; CI, confidence interval; EOT, end of treatment; HBV, hepatitis B virus; HBsAg, hepatitis B surface antigen; HBcrAg, hepatitis B core related antigen; NA, nucleoside analogue; TDF, tenofovir disoproxil fumarate.

**Table 4:** Multivariable analysis of HBsAg decline by generalized estimating equations analysis.

Post-Treatment HBsAg Decline				
HBsAg Decline	Beta	Standard Error	95% CI	*p* Value
Groups				
I	Ref			
II	−0.335	0.083	−0.499–0.172	<0.001
III	0.021	0.143	−0.259–0.301	0.886
Time (per month)	0.022	0.002	0.019–0.026	<0.001
Age (per year)	−0.007	0.005	−0.016–0.002	0.138
Sex (male vs. female)	−0.013	0.099	−0.206–0.181	0.897
TDF vs. entecavir	0.028	0.097	−0.162–0.217	0.775
HBV genotype (C vs. B)	0.138	0.101	−0.059–0.336	0.17
NA-naïve (yes vs. no)	−0.186	0.098	−0.379–0.007	0.058
GPT (per U/L)	0.00019	0.00011	−0.00003–0.00041	0.091
HBV DNA (per log IU/mL)	0.019	0.036	−0.052–0.091	0.597
Treatment duration (per week)	0.003	0.003	−0.003–0.009	0.277
Consolidation duration (per week)	−0.001	0.003	−0.006–0.004	0.667
HBsAg at initial treatment (per log IU/mL)	0.092	0.06	−0.026–0.209	0.127
HBcrAg at initial treatment (per log U/L)	−0.093	0.032	−0.156–0.03	0.004
HBsAg at EOT (per log IU/mL)	−0.162	0.056	−0.271−0.053	0.004
HBcrAg at EOT (per log U/L)	−0.082	0.051	−0.183–0.018	0.107

ALT, alanine aminotransferase; CI, confidence interval; EOT, end of treatment; HBV, hepatitis B virus; HBsAg, hepatitis B surface antigen; HBcrAg, hepatitis B core related antigen; TDF, tenofovir disoproxil fumarate.

**Table 5:** Factors associated with HBsAg loss in the patients of Groups I and III.

Variable	Comparison	Univariate Analysis HR (95% CI) *p* Value		Multivariate Analysis HR (95% CI) *p* Value	
Age (years)	Increase per year	1.015 (0.991–1.039)	0.217	-	-
Sex	Male vs. female	1.181 (0.641–2.177)	0.594	-	-
ALT (U/L)	Increase per U/L	1.000 (1.000–1.001)	0.553	-	-
Total bilirubin	Increase per mg/dL	1.004 (0.938–1.076)	0.900	-	-
HBV DNA	Increase per log IU/mL	0.867 (0.754–0.997)	0.045	-	-
HBV genotype	C vs. B	0.946 (0.562–1.591)	0.833	-	-
HBsAg at initial treatment	Increase per log IU/mL	0.630 (0.488–0813)	<0.001	-	-
HBsAg at EOT	Increase per log IU/mL	0.393 (0.305–0.506)	<0.001	0.390 (0.300–0.508)	<0.001
HBcrAg at initial treatment	Increase per log U/mL	0.828 (0.724–0.946)	0.005	-	-
HBcrAg at EOT	Increase per log U/mL	0.766 (0.589–0.995)	0.046	-	-
Treatment duration	Increase per week	1.006 (1.002–1.009)	0.002	1005 (1.001–1.008)	0.005
Consolidation duration	Increase per week	1.006 (1.003–1.010)	<0.001	-	-
Subgroups	III vs. I	0.805 (0.581–1.117)	0.195	-	-
Antiviral agents	TDF vs. entecavir	1.448 (0.840–2.496)	0.183	-	-

ALT, alanine aminotransferase; CI, confidence interval; EOT, end of treatment; HBV, hepatitis B virus; HBcrAg, hepatitis B core related antigen; HBsAg, hepatitis B surface antigen; HR, hazard ratio; NA, nucleoside analogue. TDF, tenofovir disoproxil fumarate.

**Table 6:** Clinical features of patients who had persistent virological remission without (Group I) or with (Group V) clinical relapse.

Variable Mean ± SD or N (%)	Patients with Sustained Virological Suppression (*n* = 128)	Patients with Clinical Relapse (*n* = 60)	*p* Value
Age (years)	50.7 ± 10.7	49.7 ± 10.1	0.564
Sex (male vs. female)	104:24	46:14	0.466
Entecavir:TDF	76:52	41:19	0.238
ALT (U/L)	305.8 ± 422.3	251.1 ± 365.3	0.563
Total bilirubin (mg/dL)	2.45 ± 4.46	2.19 ± 4.47	0.673
NA-naive	89 (69.5%)	41 (68.3%)	0.868
HBV DNA (log IU/mL)	4.97 ± 1.80	5.51 ± 1.39	0.039
HBV genotype	-	-	0.003
B	84 (65.6%)	52 (86.7%)	-
C	44 (34.4%)	8 (23.3%)	-
HBsAg at baseline (log_10_ IU/mL)	2.54 ± 1.03	2.83 ± 0.72	0.054
HBsAg at EOT (log_10_ IU/mL)	1.67 ± 1.05	2.58 ± 0.49	<0.001
HBcrAg at baseline (log_10_ IU/mL)	4.57 ± 1.60	4.91 ± 1.58	0.185
HBcrAg at EOT (log_10_ U/mL)	3.30 ± 0.59	3.39 ± 0.58	0.351
Treatment duration (weeks)	169.8 ± 52.6	165.7 ± 49.0	0.613
Consolidation duration (weeks)	141.9 ± 55.0	137.1 ± 47.6	0.564

ALT, alanineaminotransferase; EOT, end of treatment; HBV, hepatitis B virus; HBsAg, hepatitis B surface antigen; HBcrAg, hepatitis B core related antigen; NA, nucleoside analogue; TDF, tenofovir disoproxil fumarate.

## Data Availability

All data may be obtained from the corresponding authors upon request.
